# Cardiac CT Angiography-Guided Management of Giant Coronary Artery Aneurysms in Atypical Kawasaki Disease: A Case Report and Review of Literature

**DOI:** 10.7759/cureus.44425

**Published:** 2023-08-31

**Authors:** Jyothi Prakash R, V Rama Subramanyam Muddana, Hareesh Kumar Munugala, Krishna Shriram Dhanasekaran

**Affiliations:** 1 Pediatric Cardiology, Aster Ramesh Hospitals, Vijayawada, IND; 2 Radiology, Aster Ramesh Hospitals, Vijayawada, IND; 3 Research and Publications, Aster Ramesh Hospitals, Vijayawada, IND

**Keywords:** intravenous immunoglobulin, echocardiography, cardiac computed tomographic angiography, ccta, coronary artery aneurysm, atypical kawasaki disease

## Abstract

Kawasaki disease (KD) is an auto-immune, acute febrile illness mostly affecting young children. It may develop into vasculitis characterized by coronary artery aneurysms (CAA) if not diagnosed and managed earlier. Timely diagnosis and appropriate treatment eventually avoid the risk of the development of CAA. We present the case of a 21-month-old female child with a history of persistent fever for nearly 10 days who further developed desquamations and presented for cardiac evaluation. Atypical KD with the development of giant CAAs was effectively diagnosed by cardiac computed tomographic angiography (CCTA) and was appropriately managed.

## Introduction

Kawasaki disease (KD), or mucocutaneous lymph node syndrome, is an acute febrile illness [1]. It is an autoimmune, inflammatory vasculitis that mostly affects infants and children younger than five years of age, affecting the coronary arteries, which may result in coronary artery aneurysms (CAA) with lifelong manifestations [2]. Kawasaki disease is of unknown aetiology and is diagnosed with persistent fever for more than five days and at least four or more of its principal clinical manifestations, including erythematous changes of the lips and oral mucosa, bilateral non-purulent conjunctivitis, a nonspecific rash, erythema and oedema of the feet and hands, or periungual desquamation (sub-acute phase), and cervical lymphadenopathy (≥1.5 cm) [3,4]. Children often have atypical (incomplete) presentations of KD and are at high risk for the development of CAA [5].

We report the diagnosis and treatment of atypical KD in a 21-month-old female child who presented with persistent fever and thrombocytosis. The development of CAA was partially detected by echocardiography and completely by cardiac computed tomographic angiography (CCTA).

## Case presentation

**Figure 1:**
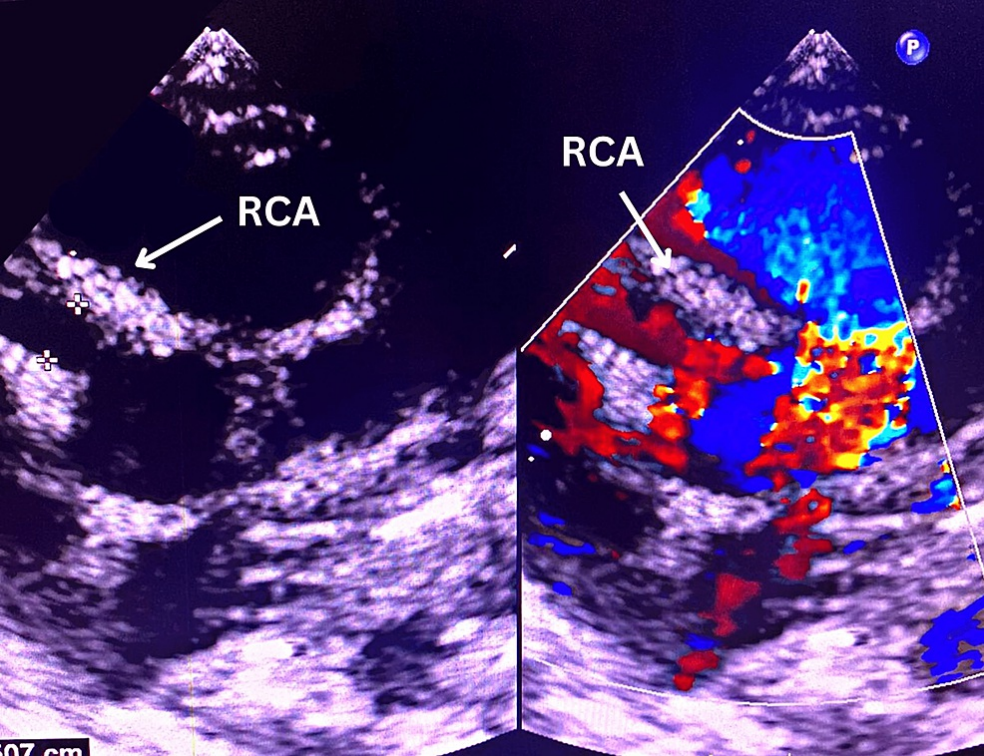
Echocardiographic image showing a coronary artery aneurysm in the RCA (arrows) RCA: right coronary artery

A 21-month-old female child, previously healthy, presented to a primary care clinic with a history of fever for 10 days with cold, dysuria, and pedal oedema. Admission workup revealed 8.8 g/dL of haemoglobin (↓ Hb) and 19,400 cells/mm3 of total leukocyte count (↑ TLC). The bacterial culture sensitivity test was sterile. Immunoglobulin G (IgG) and immunoglobulin M (IgM) were positive on dengue serology. Thrombocytosis (platelet count: 9,50,000 cells/μL) was noted on the ninth day. Desquamation development in the periungual and perianal regions was noted in the second week of illness with persistent fever. Atypical Kawasaki disease (KD) was suspected using the aforementioned cardinal manifestations and haematological reports. The child was referred to our tertiary care centre for a cardiac evaluation to rule out atypical KD. Initial screening by echocardiography noted an aneurysmal dilatation in the right coronary artery (RCA) (Figure 1).

**Figure 2:**
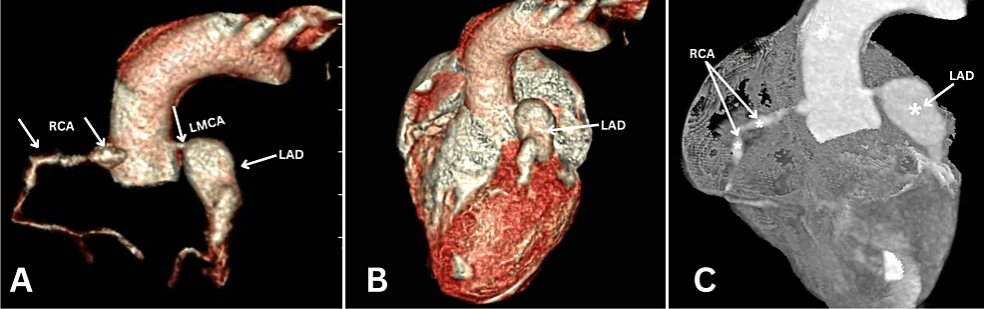
Comprehensive assessment of coronary artery involvement by CCTA: 3D VR image of (A) the aortic root with coronary vessels, (B) the whole heart, and (C) an outline MIP image of the heart, revealing several aneurysms in the proximal and mid-RCA (arrows and stars) and distal LMCA (arrows), and giant aneurysms in the proximal and mid-LAD (arrows and stars). CCTA: cardiac computed tomographic angiography; 3D: three dimensional; VR: volume rendered; MIP: maximum intensity projection; RCA: right coronary artery, LMCA: left main coronary artery; LAD: left anterior descending artery

The child was started on intravenous immunoglobulin (IVIG), low molecular weight heparin (LMWH), and aspirin. At her first review after a week, repeated echocardiography revealed the development of a new aneurysmal dilatation in the left main coronary artery (LMCA) and worsening of the previous aneurysm in the RCA. Hence, cardiac computed tomographic angiography (CCTA) was performed for a detailed diagnosis. The three-dimensional (3D) volume rendered (VR) (Figure 2A, 2B) and the maximum intensity projection (MIP) (Figure 2C) imaging analyses on CCTA revealed extensive and diffuse coronary artery dilatations, i.e., coronary artery aneurysms (CAA) in more than one vessel.

Giant CAAs (grade-3) over a length of 26 mm were noted on the distal LMCA and the proximal and mid-left anterior descending artery (LAD) (Figure 2). The proximal and midparts of the RCA were also noted to have aneurysmal dilatations of 10 mm and 8 mm in length, respectively (Figure 2).

After the CCTA, the child was therapeutically managed with a standard IVIG dose of 18 g (2 g/kg body weight), LMWH, and aspirin. The child was discharged stably following a defervescence after 24 hours of IVIG administration. The LMWH was switched to oral nicoumalone (2 mg). The parents were advised to bring her for regular follow-up for cardiac evaluation and frequent monitoring of prothrombin time (PT) and international normalised ratio (INR) to achieve the desired INR target between 2 and 2.5.

## Discussion

Kawasaki disease is an acute systemic vasculitis of unknown origin, mainly affecting children between six months and eight years of age [6]. The diagnosis is characterised by persistent fever for more than five days and four out of five cardinal features (conjunctivitis; erythematous rash; desquamation or erythema or cracking of lips, oral cavity, palms, and soles; polymorphous exanthema of the trunk; and cervical lymphadenopathy) or less than four in the case of echocardiographic or coronary angiographic findings with coronary artery involvement [3,4,6,7]. In our case, the infant had a persistent fever for a two-week period, with the aforementioned desquamations and echocardiographic and angiographic findings of CAA. Findings like decreased haemoglobin and hematocrit, leukocytosis, and thrombocytosis were noted. In our case, the echocardiographic imaging initially revealed aneurysmal dilatations, or CAA, only in the RCA.

The CCTA findings provided a detailed diagnosis of giant CAA involving distal LMCA, proximal and mid-LAD, and several aneurysms in the proximal and mid-RCA. Echocardiography is the first-line imaging for the evaluation of coronary artery involvement in KD, as recommended by the American Heart Association (AHA) [8,9]. Nevertheless, echocardiography is highly operator-dependent, and the available acoustic windows limit the evaluation of the mid-and distal coronary artery segments [10]. However, CCTA is sensitive and specific in depicting even small aneurysms [11].

The CCTA imaging in KD has been performed for a detailed evaluation of coronary artery involvement when the echocardiographic images are unclear or it is difficult to locate the aneurysm. It is mostly used in both acute and chronic phases of KD. Newer generation computed tomographic (CT) scanners provide detailed information on the arterial location, size, structure, and number of CAAs with improved spatial resolution and better image quality [2,12,13]. As per the technical advancements in imaging, the radiation dose used was significantly lower for imaging with a 256-slice CT scanner at a heart rate (HR) of 132 bpm. An intravenous (IV) contrast medium of 8 ml (Iohexol 350 mg) was injected manually, and 3D post-processing was performed.

In our case, the fever reduced following an IVIG infusion. The infant was switched from LMWH to oral nicoumalone on discharge, advising a follow-up. Similarly, an infant treated by Dodi et al. [14] received an IVIG infusion (2 g/kg) in a single dose for 12 hours, and aspirin (100 mg/kg in four doses) was immediately started with LMWH. The clinical status improved rapidly, with a significant decrease in inflammatory markers and platelet count. A complete normalisation 20 days after the IVIG infusion was observed. The LMWH was then switched to warfarin [14]. A delay in diagnosis with incomplete clinical manifestations mostly leads to the development of CAA.

In children, a persistent fever for more than five days should be suspected of Kawasaki disease (KD). Despite the presentation of fewer classical symptoms, a delayed diagnosis could be avoided by a combined evaluation of inflammatory markers and coronary artery involvement. Atypical presentations always need aggressive management with IVIG, steroids, or infliximab (5 mg/kg). Timely diagnosis and appropriate treatment prevent the development of serious coronary artery aneurysms in Kawasaki disease (KD).

## Conclusions

Early recognition and prompt initiation of appropriate therapies are key factors in managing KD to prevent CAA and other potentially life-threatening complications. By employing advanced imaging techniques like CCTA, clinicians can accurately assess coronary artery involvement and tailor treatments to improve affected children's long-term prognosis and overall well-being. Further research and awareness are essential to better understanding and effectively managing this challenging vasculitis.
